# Can we Build on Social Movement Theories to Develop and Improve Community‐Based Participatory Research? A Framework Synthesis Review

**DOI:** 10.1002/ajcp.12142

**Published:** 2017-05-04

**Authors:** Marie‐Claude Tremblay, Debbie H. Martin, Ann C. Macaulay, Pierre Pluye

**Affiliations:** ^1^ Department of Family Medicine and Emergency Medicine Office of Education and Continuing Professional Development Université Laval Québec QC Canada; ^2^ Faculties of Health Professions and Dentistry Dalhousie University Halifax NS Canada; ^3^ Department of Family Medicine McGill University Montreal QC Canada

**Keywords:** Community‐based participatory research, Social movements, Collective action, Process evaluation, Program evaluation, Health promotion

## Abstract

A long‐standing challenge in community‐based participatory research (CBPR) has been to anchor practice and evaluation in a relevant and comprehensive theoretical framework of community change. This study describes the development of a multidimensional conceptual framework that builds on social movement theories to identify key components of CBPR processes. Framework synthesis was used as a general literature search and analysis strategy. An initial conceptual framework was developed from the theoretical literature on social movement. A literature search performed to identify illustrative CBPR projects yielded 635 potentially relevant documents, from which eight projects (corresponding to 58 publications) were retained after record and full‐text screening. Framework synthesis was used to code and organize data from these projects, ultimately providing a refined framework. The final conceptual framework maps key concepts of CBPR mobilization processes, such as the pivotal role of the partnership; resources and opportunities as necessary components feeding the partnership's development; the importance of framing processes; and a tight alignment between the cause (partnership's goal), the collective action strategy, and the system changes targeted. The revised framework provides a context‐specific model to generate a new, innovative understanding of CBPR mobilization processes, drawing on existing theoretical foundations.

## Highlights

- Based on framework synthesis, we propose a conceptual framework to understand CBPR processes.
- Drawing on social movement theories, this framework emphasizes:
  – The central and catalytic role of the partnership in the mobilization process;
  – Framing processes, i.e., construction of shared interpretation of the partnership's goal and action;
  – Resources and opportunities as necessary components feeding the partnership's development.
- Our framework provides innovative insights for the implementation and evaluation of CBPR.

## Introduction

Community psychology, community development, social work, public health, and health promotion are fields of action and research that aim to transform the life and health conditions of individuals, groups, and populations (O'Neill & Stirling, 2006; Perkins & Zimmerman, 1995; Rappaport, 1981, 1987; Rootman, Goodstat, Potvin & Springett, 2001). These fields have a vested and inherent focus on social change (Lehrner & Allen, 2008), and build on approaches that emphasize critical investigation, empowerment, as well as transformative action (Maton, 2000; Minkler & Wallerstein, 1997). Community‐based participatory research (CBPR) is one of these approaches, viewed as a valuable way to empower people and groups, enhance their voice and power in society, and facilitate social change (Cargo et al., 2008; Green & Kreuter, 2005; Labonte, 1994; Maton, 2000; Merzel & D'Afflitti, 2003).

Community‐based participatory research is an approach to research that engages community and academic partners in a common knowledge production process aimed at understanding and improving the well‐being and health of groups and communities (Castleden, Morgan & Lamb, 2012; Green et al., 1995; Horowitz, Arniella, James & Bickell, 2004; Israel et al., 2003; Schultz, Collie‐Akers, Fernandez, Fawcett & Ronan, 2009). From an epistemological standpoint, CBPR involves questioning the nature of knowledge and its underlying structures; it legitimizes experiential knowledge and assumes that, by understanding and partaking in the practices of those involved, partners can improve their situation and balance power relationships (Baum, MacDougall & Smith, 2006). Thus, CBPR is anchored in the development of relationships of respect and trust that foster power sharing between researchers and those researched in all phases of the research process (Baum et al., 2006). In so doing, CBPR also implies balancing research with positive and tangible social change for the mutual benefit of all partners (Castleden et al., 2012; Israel et al., 2003; Mohatt, 2014). Recent reviews point to benefits of CBPR, including increased sustainability of project goals, the creation of unanticipated and spin‐off projects, and the generation of policy changes and systemic transformations (Jagosh et al., 2012, 2015).

Community‐based participatory research is inspired by social change theories, such as Freire's critical epistemology which views action and critical reflection as indissolubly united, a “praxis” that fosters creativity, critical consciousness, and transformational changes (Baum et al., 2006; Freire, 2000). In the context of CBPR, this praxis provides a broad frame in which to envision research as a process of engagement and cooperation that acknowledges power relationships, and highlights the necessity to enter into critical dialog with the community and address issues that are important to the lives of its members (Freire, 2000; Hall, 2005). Freire's praxis proposes an important role for leaders, and emphasizes, among others, the principles of cooperation and community organization (Freire, 2000).

A long‐standing challenge of CBPR has been to anchor practice and evaluation in a relevant and comprehensive theoretical framework of community change, one that links the processes of action and intervention to targeted changes across multiple levels of influence, including the broader social context (Cargo & Mercer, 2008; Fawcett, Schultz, Watson‐Thompson, Fox & Bremby, 2010; Jagosh et al., 2012; Merzel & D'Afflitti, 2003; Wallerstein et al., 2008). Typically, conceptual frameworks for CBPR have focused on developing practical implementation and outcome benchmarks (Fawcett et al., 1995) or describing group dynamics (inside the partnership itself) that lead to successful collaborations (Schulz, Israel & Lantz, 2003; Wallerstein et al., 2008). However, these frameworks generally lack a comprehensive theoretical explanation of the dynamic processes that lead to community mobilization and change in the context of CBPR (Merzel & D'Afflitti, 2003). Building on a systematic review of 32 community‐based programs, Merzel and D'Afflitti (2003, p. 557) stress that “Although community participation and multilevel ecological models provide useful frameworks for addressing community health issues, there is a need to improve understanding of the precise ways in which these models are operationalized and influence program outcomes.” Adapted from the Institute of Medicine's framework for collaborative public health action (Committee on Assuring the Health of the Public in the 21st Century, 2003). Fawcett et al.'s (2010) sequential and interactive framework constitutes a notable effort in this regard and appears to be more inclusive than other frameworks in terms of integrating change levels and processes. This framework, which aims at providing guidance on different processes for collaborative action in communities, has five main components: (a) assessment and collaborative planning (analyzing information, establishing a vision, developing a logic model, and strategic plans); (b) implementing targeted action (defining an operating structure and mechanisms, developing leadership and community mobilization); (c) changing conditions in communities and systems (implementing interventions and assuring assistance); (d) achieving widespread change in behaviors (documenting progresses); (e) improving population health and health equity (documenting outcomes and sustaining the work; Fawcett et al., 2010). However, this framework does not take into account the broader context of action and external inputs, and proposes only a general representation of community mobilization.

Social movement theories are a well‐developed body of theories that can be used to inform the development of a coherent and unifying framework of community change processes for CBPR. Social movement theories examine the conditions under which collective action emerges and develops to promote social change around a specific issue, and provide a range of analytical tools that help understand and facilitate these processes. On account of their transformational and empowering potential, social movement theories have attracted much interest in fields aimed at social betterment through participation, such as community psychology or health promotion (Maton, 2000, 2008; Minkler & Wallerstein, 1997; Munger, MacLeod & Loomis, 2016; Nutbeam, 1998; Tesdahl & Speer, 2015). For instance, in community psychology, social action and social movement organizations have been proposed as empowering settings, with the potential to promote community betterment by fostering citizen mobilization and social change (Maton, 2008). In health promotion, authors such as Nutbeam (1998, p. 38) have emphasized the potential of social mobilization to address “some of the underlying social and economic determinants of health which require sustained activism, and to offer greater opportunity for community control and empowerment.” However, these theories have not been used to inform the development and implementation of CBPR, and there remains a need to provide a coherent theoretical explanation of how mobilization is fostered and leads to systemic changes in the context of a CBPR project (Fawcett et al., 2010).

Drawing on a framework synthesis (Carroll, Booth & Cooper, 2011; Carroll, Booth, Leaviss & Rick, 2013; Dixon‐Woods, 2011; Oliver et al., 2008) of key CBPR projects, *this study aims to describe the development of a multidimensional conceptual framework building on social movement theories capable of drawing out identifiable elements of CBPR processes*. In this work, we favored a specific rather than exhaustive search strategy, focused around the need to find information‐rich examples of illustrative CBPR. We believe that using a social movement conceptual framework to understand and conceptualize community change processes will provide interesting and innovative insights for the implementation, improvement, and evaluation of CBPR initiatives.

## Literature Review

### Key Concepts Relating to Social Movements

The term *social movement* is often used to describe a broad range of social transformations in a number of fields, leading to the proliferation of definitions and descriptions. Traditionally, definitions of social movements have highlighted the noninstitutionalized and minimally organized nature of collective actions which form around specific *grievances* (discontent) in order to promote—or resist—social change (Jenkins, 1983; Tilly, 1978; Wilkinson, 1971). In fact, “social movements (…) can be thought of as organized yet informal social entities that are engaged in extra‐institutional conflict (…) oriented towards a goal. These goals can be either aimed at a specific and narrow policy or be more broadly aimed at cultural change” (Christiansen, 2009, p. 2). Social movements represent a “societal level force” for groups in quest of social justice and empowerment (Maton, 2000, p. 35). Recent approaches to the study of social movements have considered these efforts as extensions of institutionalized action that seek to promote personal, cultural, or institutional change (Gamson, 1992; Goodwin & Jasper, 1999; Jenkins, 1983; Jenkins & Perrow, 1977).

Social movement has given birth to many theoretical approaches, such as class conflict, collective behavior, value‐added theory, political process theory, resources mobilization theory and framing theory, among others (Horn, 2013; Jenkins, 1983; McAdam, McCarthy & Zald, 1996; Mueller, 1992; Tilly, 1978). These various theoretical lenses can be attributed to shifting theoretical understandings among academics, but have also emerged through the analysis of new forms of social mobilization (Horn, 2013). For instance, stemming from an analysis of the 1960s' movements analysis, *resources mobilization theory* is a strain of social movement theories that emphasizes the importance of resources in promoting social change (Jenkins, 1983; Jenkins & Perrow, 1977; McAdam et al., 1996; Oberschall, 1973). In this theoretical perspective, the formation and mobilization of movements is dependent on changes in resources, group organization, and opportunities for action (Jenkins, 1983). Movements are also assessed based on their capacity to garner and use resources to bring about change. Resources are viewed as tangible or intangible assets brought by groups and individuals within the movement, and they play an important role in shaping the capacity of the movement to reach its goal (Freeman, 1979; Horn, 2013). This approach also posits a strong organizational base: social movements are viewed as catalyzed by (pre‐existing or newly created) organizations involving leaders or spokespersons, members or followers, who build the movement by mobilizing efforts and organizing resources to bring about collective action (Jenkins, 1983). Whereas the principal resource of a movement is the voluntary labor of its members, resource mobilization also involves developing and sustaining their participation in service of the movement's goal (Tesdahl & Speer, 2015). Therefore, members are generally considered as a defining element of social movements; through their mobilization and organization, they give meaning and carry the movement (Horn, 2013).

*Political process theory*, a critique of the resources mobilization approach, outlines the importance of political contexts and opportunities in the emergence and development of social movements (Goodwin & Jasper, 1999; Horn, 2013; McAdam et al., 1996; Mueller, 1992). Here, social movements are seen as developing dynamically in response to contingent opportunities (viewed as political structural changes and power shifts) that influence their efforts to mobilize members and resources (Goodwin & Jasper, 1999; Meyer & Minkoff, 2004; Pichardo, 1997). From this perspective, some contexts are more conductive to social movement activities, and to leveraging political opportunities (Meyer & Minkoff, 2004). These opportunities may include political instability resulting from conflict between elites and increased access to elite allies or political decision‐making process. Political process theory also outlines the importance of the collective action strategy, which informs the movement's theory of change; i.e., the way it intends to reach its goals, considering these particular opportunities (Meyer & Minkoff, 2004).

More recent strains of social movement theories have tried to engage elements of social psychology to integrate an “individual agency” aspect to the creation and action of a movement (Goodwin & Jasper, 1999; Horn, 2013; Jasper, 2004). For instance, *framing theory*, developed in the 1970s and 1980s, highlights the importance of collectively shared interpretations and understandings—frames—that the movement develops to successfully mobilize individuals around a sense of moral struggle (Benford & Snow, 2000; De la Porta & Diani, 2006; Gamson, 1992). In this approach, framing is the active process of constructing shared interpretations, representations, and meanings of social situations and issues (Snow & Benford, 1988). As all participants have different frames, there is a need to “align” individual frames to make individual interests, beliefs, and values congruent with the activities, ideas, and goals of the movement (Snow, Burke Rochford, Worden & Benford, 1986). Collective action frames are constructed through negotiation among movement adherents to identify a condition or situation they believe is in need of change, to articulate a solution, and to motivate others to take action (Benford & Snow, 2000). Framing processes emphasize the importance of meaning making and involve the redefinition of unquestioned social phenomena, producing alternative understandings of taken for granted situations (Lehrner & Allen, 2008; Maton, 2008). These processes lead to the definition of a common cause, a central vision that guides action and federates members (Horn, 2013).

At the same time, it is important to view social movements from a diachronic perspective, as phenomena that develop and evolve over cycles of time (De la Porta & Diani, 2006; Masters & Osborn, 2010). Although the lifecycle of a social movement is defined differently depending on the particular theoretical approach used, four broad stages are generally distinguished in the literature. The *first stage* is the emergence stage, representing the construction of the infrastructure of the movement (e.g., broad base of activist members, networks, organizing centers) in response to a general discontent over an issue. In the *second stage*, the identity and vision of the movement are developed around a clear interpretative discourse, as the movement becomes more organized and strategic. The *third stage* is sometimes labeled “the movement's moment” (Masters & Osborn 2010) and is a transformative stage through which the movement implements its collective action. During this stage, the movement benefits from political power and a strong level of organization to progress toward its goal. In the *fourth stage*, the social movement declines or consolidates, as the movement fails and dissipates, or achieves its goals and sees its results institutionalized and sustained (Horn, 2013; Masters et al., 2010). This process of development is not linear: “As movements form they go through stages of growth and change, in some cases growing systematically in strength and impact over time and in others fluctuating in response to internal dynamics and external pressures.” (Horn, 2013, p. 19).

### Conceptual Framework

**Figure 1:**
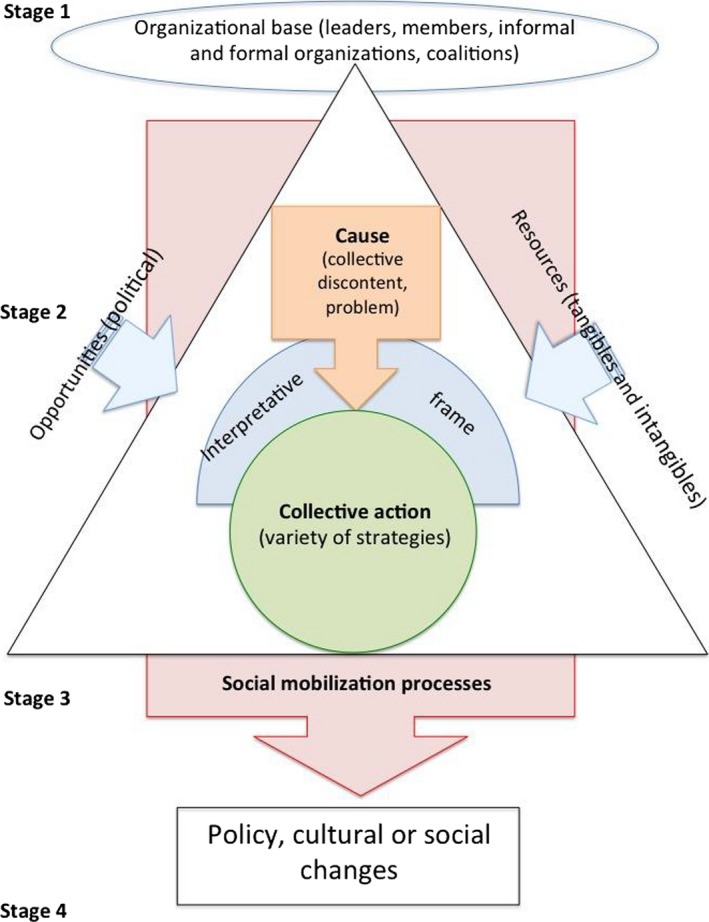
The initial conceptual framework represents a graphic summary of key concepts of social movement theories and their relationships.

Although the aforementioned theoretical perspectives emphasize different elements of social movements, most representations highlight similar characteristics. Key characteristics of social movements that are foundational to the conceptual framework used in this study (Fig. 1) can be summarized in seven points (De la Porta & Diani, 2006; Horn, 2013; Masters & Osborn, 2010), namely, that social movements:

1. Rely on a strong organizational base (involving leaders, members or followers, formal or informal organizations, and coalitions) to build and organize the movement;
2. Pursue a political agenda or a “common cause”;
3. Engage in collective actions that are oriented toward clear targets, and use a variety of strategies in pursuit of their goals;
4. Use interpretative frames to define a problematic situation in need of change, to articulate a solution, and to raise awareness or motivate others to take action or garner support;
5. Develop in relation to specific opportunities and follow a long lifecycle that retains some continuity over time;
6. Build on tangible and intangible resources from individuals and groups; and
7. Seek policy, social, or cultural changes.

### Relevance of Social Movement Theories to CBPR

Social movements and CBPR present interesting similarities, which outline the potential of the former to understand the latter. For instance, both social movements and CBPR emerge from a collectively shared concern, problematic situation, or social condition identified by the community (Israel et al., 2003). “A CBPR approach begins with the goal of addressing a community‐identified social problem (often health‐related), and at its base is a commitment to researching issues that matter in people's lives” (Letiecq & Schmalzbauer, 2012, p. 247). In addition, and as is the case for social movements, CBPR includes an important action component to promote change (Ingram et al., 2015). This requires a deep investment in social transformation, one that involves challenging the *status quo* to improve the lives of community members (Israel et al., 2003; Velasquez, Knatterud‐Hubinger, Narr, Mendenhall & Solheim, 2011), which is why CBPR's action is sometimes viewed as an inherently political and moral endeavor to “pursue social change and justice through a process of grassroots democracy, organizing, and shifting power relations” (Letiecq & Schmalzbauer, 2012, p. 247). Furthermore, both social movements and CBPR need a certain level of organization and build on community members, resources, and assets to carry out their functions and goals (Israel et al., 2003). Ultimately, both aim to be sustainable, through the development of long‐term efforts and sustainability objectives (Horn, 2013; Israel et al., 2003). More importantly, both CBPR and social movements have been envisioned as following a four‐phase developmental process involving engagement, formation, implementation, and maintenance (Cargo & Mercer, 2008; Kreuter, Lezin & Young, 2000).

Social movements and CBPR seek to reverse unequal relations of power by creating social, policy, and broad systemic changes (Cargo & Mercer, 2008; Israel et al., 2003; Velasquez et al., 2011). This is not to say that the two phenomena are the same. In fact, social movements are traditionally seen as having a greater geographic scope than a single or a set of communities. In addition, social movements are generally considered as operating in opposition to the prevailing system, whereas CBPRs are often “embraced by government policy” and funded, for the most part, by it (Labonte, 1994, p. 245). Finally, CBPR is an approach to action and research that is systematically grounded in empirical science, which is not necessarily the case for social action (Munger et al., 2016). Nonetheless, both processes share similar outlines and goals, owing to CBPR's roots in popular education and emancipation traditions (Baum et al., 2006; Cargo & Mercer, 2008; Castleden et al., 2012).

## Methods

### General Approach

The general methodological approach of this study is based on framework synthesis. This recent type of review, adapted from framework analysis—a technique for data analysis in primary qualitative studies (Dixon‐Woods, 2011) —, is a highly structured approach to organize qualitative data, based on a priori themes drawn from a conceptual framework (Carroll et al., 2011, 2013; Dixon‐Woods, 2011; Oliver et al., 2008). While framework synthesis is largely a deductive approach, it also includes an inductive component building on thematic analysis to identify potential new themes from the data, binding the strengths of both deductive and inductive approaches (Carroll et al., 2013; Dixon‐Woods, 2011; Oliver et al., 2008).

Framework synthesis operates in four general steps. The first step consists in identifying a theory/model/framework relevant to the phenomenon of interest, which can come from background material, consultation, team discussion, or the literature. The most meaningful components of the framework are reduced into a priori themes, concepts, or categories that are used to analyze data. The second step is the identification and selection of primary studies to be included in the review following conventional literature review methods. The third step consists in analyzing information from the included papers, based on the a priori themes from the initial framework. This step also uses thematic analysis, according to Miles and Huberman techniques for primary data (Miles & Huberman, 1994), to generate new themes that may be incorporated as they emerge. The fourth step of framework synthesis involves recreating and recombining the themes into a refined framework outlining the nature, dimensions, and relationships between the themes. The product of framework synthesis can take the form of a chart of the key dimensions of the studied concepts, which may be used to map the nature and range of these dimensions, and to find associations between concepts. Details of the steps we followed in this study are described below.

### Step 1: Identifying the Initial Conceptual Framework

Consistent with the first step of framework synthesis, we sought a relevant framework to capture CBPR's processes and outcomes. We consulted major work in the theoretical literature and seminal work on social movements to guide the development of a conceptual framework that could be used to investigate mobilization processes in the context of CBPR. A sociology expert helped identify seminal work in social movement theories. We also identified peer‐reviewed literature using the reference lists and online searches. Given the multitude of theories proposing an explanation of social movement mobilization processes, we employed an iterative process, familiarizing ourselves with the literature on social movement theories, and gradually developing our conceptual framework based on the main concepts derived from these theories and relationships between them. Once finalized, the initial framework was validated by consensus with the research team. Key concepts of social movement theories and their relationships are represented in our initial conceptual framework, from which a priori themes have been extracted to form the analysis grid (Table 1).

**Table 1:** Description of the a priori themes from the initial conceptual framework

Themes/Categories	Descriptions
Organizational base	Organizations instrumental to the movement's creation and collective action (involving organizations, coalitions, leaders or spokespersons, members)
Cause	Agenda of the movement formalized in a framing discourse around a collective discontent
Collective action strategy	General action strategy used by the movement, targets, and level of action (policy, organizations, individuals)
Interpretative frame	In negotiation among movement adherents, collectively constructed frames to define a problematic situation in need of change, to articulate a solution, to raise awareness or motivate others to take action or garner support, and to demobilize antagonists
Opportunities	Structural changes and power shifts (mostly political) that are crucial to a movement's creation, infrastructure building, and resources mobilization
Resources	Tangible and intangible assets used by the movement to carry out its action, brought by organizations and individuals
Policy, social, or cultural changes	Changes achieved as a result of the movement's action, also include new capacities and new possibilities of action for groups and people engaged
Stage 1	Emergence: Beginning of the movement and building of movement's infrastructure in response to a general discontent over an issue
Stage 2	Coalescence: Development of the movement's identity and vision, the movement becomes more organized and strategic
Stage 3	The movement's moment: Implementation of the movement's collective action, the movement shows a high political power and a strong level of organization
Stage 4	Decline or consolidation: The movement fails and dissipates, or achieves its goals and sustains itself

### Step 2: Developing a Search Strategy

#### General Consideration

This framework synthesis is part of a larger project aimed at analyzing the intermediate community‐level outcomes of a CBPR project developed with an Indigenous community in Canada. In this view, the search strategy was not exhaustive but rather was articulated around the need to find information‐rich examples of illustrative CBPR projects that could be used to develop and refine a relevant conceptual framework for the larger research project. Thus, the term “community‐based participatory research,” principally used in North America, was preferred over such terms as “participatory action‐research.”

#### Eligibility Criteria

We sought detailed descriptions of CBPR projects presenting typical characteristics, in accordance with Israel et al. (2003). These include focusing on the community as the main unit of identity; presenting an equitable partnership between all project partners throughout the process; showing a balance between the research and action components for the mutual benefit of all partners; challenging social determinants of health by promoting system and policy changes; and promoting long‐term engagement and sustainability of the efforts. The eligibility criteria for selecting bibliographic records and full‐text papers were developed in question format according to these considerations (Table 2). Although the analysis was meant to focus on qualitative descriptions of projects in the included papers, qualitative as well as quantitative papers were included if they met the selection criteria, which included providing a sufficient description of the intervention's processes and development.

**Table 2:** Eligibility criteria

Identification criteria of bibliographic records	Selection criteria of full‐text papers
1. Does the citation indicate primary participatory research?	1. Does the full‐text paper describe a participatory project showing high level of involvement/partnership with non‐academic partners, in at least two phases of the research, such as: (a) identifying or setting the research questions; (b) setting the methodology or collecting data or analyzing the data; (c) uptake or dissemination of the research findings.
2. Does the citation indicate a health‐related intervention component to the research?	2. Does the full‐text paper still indicate a health‐related intervention/action component, in balance with the research component? (i.e., An intervention component aiming to promote or improve health has to be an integral part of the research)
3. Does the citation indicate a community setting?	3. Does the full‐text paper still describe a community setting and community change targets? (i.e., A project aiming at environmental and system changes in the community)
4. Does the citation indicate some form of description or information about the CBPR process?	4. Does the full‐text paper describe a sustainable project, involving long‐term commitment from the partners and sustainability aspects such as spinning‐offs or institutionalization of effective strategies, incorporation or successful fund raising after the project?
5. Does the citation indicate a paper in English?	5. Does the full‐text paper provide enough description of the participatory research process or development?

#### Information Sources and Search Strategy

Studies were identified by searching four major scientific health‐related databases: Pubmed, Embase, Cinahl, and Psychinfo from inception to August 1st, 2015. The search was conducted by a professional health librarian using “Community‐based participatory research” as a keyword (in title).

#### Study Selection Process

Using Endnote X7 (Thomson Reuters, 2013), the first author created a database and screened title and abstracts of identified papers according to eligibility criteria. As we sought detailed descriptions and primary studies, we excluded letters to the editor, editorials, reviews, and papers presenting more than one example of CBPR. Guided by the health librarian, the principal author retrieved the identified full‐text papers and reviewed them according to selection criteria, using a table developed in Excel (Microsoft Corporation, 2010).

Given that CBPR projects are often described across several publications, the first author then searched for companion and project‐related documents for each included study. Between September 1st and October 1st, 2015, the first author performed: (a) backward and forward citation tracking in Scopus; (b) Google searches using the project or partnership's name to find official project Websites; and (c) project Website searches to locate documents, such as reports and other gray literature. We purposely excluded newspaper articles from this category, as we wanted to limit and concentrate the analysis on official and primary sources of information. We then contacted principal authors of the main papers by email and asked them for supplemental documents and papers about the CBPR projects.

### Step 3: Data Analysis

In line with the framework synthesis approach, we analyzed the information from the included projects, based on the a priori themes from the initial framework. We also used thematic analysis to generate new themes (categories) for material that the initial framework did not accommodate (Carroll et al., 2013).

The first author started by creating a database in QSR NVivo 11 (QSR International, 2015) comprising full text of main and companion papers. Based on the a priori themes of the initial conceptual framework, she developed a coding grid, which was pilot tested on two randomly selected papers, and then further refined. The first author started by extracting generic information about each of the selected CBPR projects, such as details of the partnership, setting, and intervention. Then, sentence‐by‐sentence coding was performed to assign text to one or many specific theme(s) of the coding grid (e.g., organizational base; cause; interpretative frames; collective action strategy). As previously described, this stage also involved thematic analysis on material that could not be accommodated by the coding grid, but appeared relevant to community mobilization processes. The analysis was iterative, with back and forth movements between text and coding to refine, develop, and relate themes.

We complemented and validated the analysis by conducting qualitative interviews with one of the principal authors of each of the eight main papers – this process resulted in six interviews because one of the participants was a main author/significant contributor for three of the papers. Participants were contacted by a research associate at the beginning of October 2016 and invited to participate in a short phone interview. Participants were mainly researchers (four of six) who had participated in the implementation and evaluation of the CBPR project included; the latter two were community partners who took part in the implementation of the project and collaborated with researchers. The interviews ranged in length from 20 to 50 min, and were conducted from October 24th 2016 to November 16th 2016 by the research associate. These interviews, which were structured around a preliminary summary of the key elements of CBPR projects, aimed to deepen and validate the analysis. For each theme of the framework (e.g., opportunities, resources, partners), we asked participants if the summary provided for their project was accurate and relevant, if anything was missing or should be added. In general, participants found the summaries accurate, but most added some information that was not found in the project publications and that we included in the results. They also provided new insights on the framework and our analysis, some of which are presented in the discussion.

### Step 4: Refining the Conceptual Framework

The fourth step of framework synthesis involved modifying and recombining the themes to produce a refined framework that synthesized the nature and dimensions of the themes as well as the relationships among them. This final framework represents fundamental elements of community change processes in the context of CBPR and provides a refined representation of each theme and their relationships.

## Results

### Search Results

**Figure 2:**
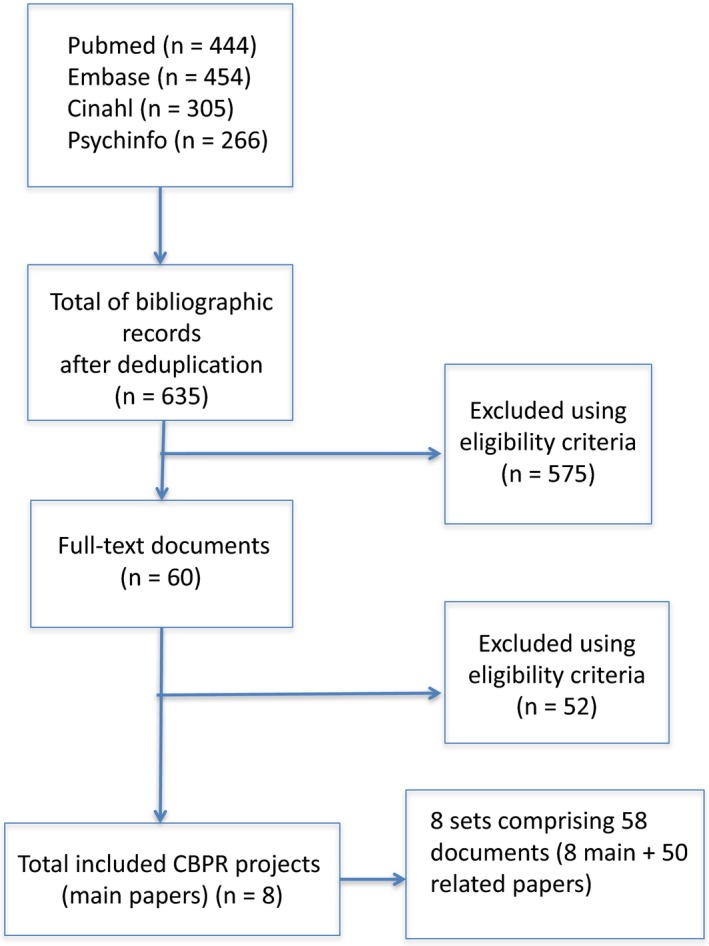
The search yielded 635 potentially relevant bibliographic records after de‐duplication. Following screening, 60 records met our criteria. Full‐text screening reduced the pool to eight articles, corresponding to eight specific CBPR projects. With companion papers and project‐related documents, the final dataset consisted of 58 documents.

The search yielded 635 potentially relevant bibliographic records after de‐duplication. Following screening, 60 records met our criteria. Full‐text screening reduced the pool to eight papers, corresponding to eight specific CBPR projects that were included in this synthesis (see Fig. 2). We found an official website for six of the eight projects. With companion and project‐related papers, the final dataset consisted of 58 documents. Generic information about each included project is presented in Appendix 1 (available online).

The eight projects were completed in the United States, with five carried out in the state of California. This may be reflective of our choice of keyword. That is, the term “community‐based participatory research” is primarily used in North America, including a major CBPR center in Berkley, California. The eight projects encompassed a great variety of partnerships: they were implemented in rural (Jernigan, Salvatore, Styne & Winkleby, 2012) and urban settings (Vasquez, Minkler & Shepard, 2006) and involved grassroots community members (Cheatham‐Rojas & Shen, 2003) or already well‐established community organizations (Fawcett, Collie‐Akers, Schultz & Cupertino, 2013). One was conducted in partnership with an Indigenous community (Jernigan et al., 2012), whereas two capitalized on youth as agents of change (Cheatham‐Rojas & Shen, 2003; Vasquez et al., 2007).

### Framework Synthesis Results

The analysis resulted in a table presenting each theme with distilled summaries from all relevant projects (Appendix 2, available online). This table was used to map each theme's key properties and dimensions in a CBPR context (Table 3). It is noteworthy that the material extracted from the eight projects supported all concepts of the initial framework, and this despite the fact that none of the CBPR projects had been built explicitly on social movement theories. However, the analysis led to the revision of some a priori themes to accommodate the context of CBPR: partnership (organizational base), interpretative frame (framing processes); and policy, cultural, and social changes (system and community changes). Also, through our analysis, we created two new themes: context and problem. The revised and new themes can, for the most part, be attributed to differences in the phenomena covered in the a priori and reviewed frameworks (social movements vs. CBPR projects). All final themes and their dimensions are described in detail in Table 3.

**Table 3:** Description of final themes

Themes/Categories	Description	Dimensions, examples
Context	Aspects of the context that play a crucial role in the emergence of the problem, the availability of opportunities and resources from which the partnership form and develop, as well as in framing processes	Aspects of context: Social context Political context Historical context Economical context
Problem	A concerning and pre‐existing health or social problem that is experienced by the community, gives birth to and justifies the partnership	Range of problems: General health status Specific health condition or disease Problematic health behaviors Problematic health determinants or exposure
Partnership	A formal partnership between academic and community partners that plays a central and catalytic role in the mobilization process, often with the addition of other partners and the community at large in the action phase	Types of partners: a researcher or a group of researchers; a pre‐existing community organization; a community–academic research organization; a coalition of organizations; a local health department; a group of grassroots community members.
Cause	Programmatic goal of a partnership, enclosing a representation of the problem, strategically and collaboratively defined to reach and mobilize community members	Range of causes: To reduce the incidence or prevalence of a specific disease or health condition; To act on an health‐deleterious situation; To promote health generally
Collective action strategy	A general line of action followed by the partnership to accomplish or achieve its goal	Levels of collective action: Systemic/environmental: To address social, physical, institutional, and political determinants of health or specific health conditions; Individual: To address individual determinants of health or health conditions (behaviors, knowledge, beliefs)
Framing processes	Collaborative and strategic interpretative construction processes that define the cause of the partnership, raise awareness of the cause in the community, and define an action to address the problem	Roles of the framing process: Define the cause of the partnership Raise awareness of the cause Define a collective action Health as a complex issue; Health as a political issue; Health as a structural issue; Health as a social/environmental justice issue
Opportunities	Temporal and contextual circumstances that have prompted the partnership's formation and building	Types of opportunities: Internal opportunities Former relationships or collaboration between the partners; External opportunities: Funding opportunities
Resources	Assets acquired and used by the partnership to carry out its function	Types of resources: Intangible resources Expert, technical, professional skills, and knowledge; Research conducted by the partnership and study results; Previous experience of the problem, the community and the local context; Pre‐existing networks and relationships; Credibility of partners; Local assets of the community; Tangible resources Funding
System an community changes	As a direct outcome of the partnership's work, changes in the social, policy, and physical environments of the community	Types of system/environmental changes: Social changes, including capacity building and empowerment Physical environment changes Policy changes
Stage 1	Creation of the partnership, with the specific aim of working on a pre‐existing health or social issue. Sometime involves research to document the issue. Implies building on tangible and intangible resources, internal and external opportunities provided by the context.	
Stage 2	Definition of the cause and development of a collective action strategy in view of particular objectives (system changes) and according to research results. Framing processes to define the cause, raise awareness of the cause in the general population, and mobilize further partners and community members in defining or validating an acceptable collective action, taking into account the particular context of the community. Sometime involves research to guide the action.	
Stage 3	Implementation of the collective action strategy, with the help of partners for action, in view of objectives and targeted system changes.	
Stage 4	Continuity of the partnership's action after it has achieved its goal or after the formal end of the partnership (forming a new incorporated organization, incorporating the partnership's priority activities into partner organizations' program, scaling up the implemented program to other levels of action with different partners, furthering participation of the community partners in similar initiatives at higher levels of action).	

### Themes

#### Context (New Theme)

The social, political, historical, and economic context of action emerged as an important theme in CBPR mobilization processes. Contexts define the way power relationships are entrenched in social structures and the situations and life conditions that community members are experiencing. This theme, which is also relevant to social movements, was not included in the initial framework because of its strong links to opportunities and resources, which were initially deemed to be broad enough to characterize the conditions linked to CBPR development. Although partly overlapping with the theme “opportunity,” context is more encompassing and takes into account opportunities, i.e., the specific circumstances that lead to the emergence and development of the partnership. Contexts do not necessarily prompt change; rather they form the setting for social situations, problems, opportunities, and resources. From our analysis, contexts play a crucial role in the emergence of the problem to be addressed by the partnership, and in the availability of opportunities and resources from which the partnership forms and develops. For instance, in the West Oakland Environmental Indicators Project (WOEIP), the fact that West Oakland is surrounded by freeways and the Port of Oakland, and was historically the final stop on the Transcontinental Railroad, is instrumental in the emergence of the problem, i.e., air pollution‐related morbidity in the community (Gonzalez et al., 2011). Moreover, the social context of the community and its history of social activism fueled local advocacy organizations, such as this project's community partner, to take action and leverage resources (Gonzalez et al., 2011).

**Figure 3:**
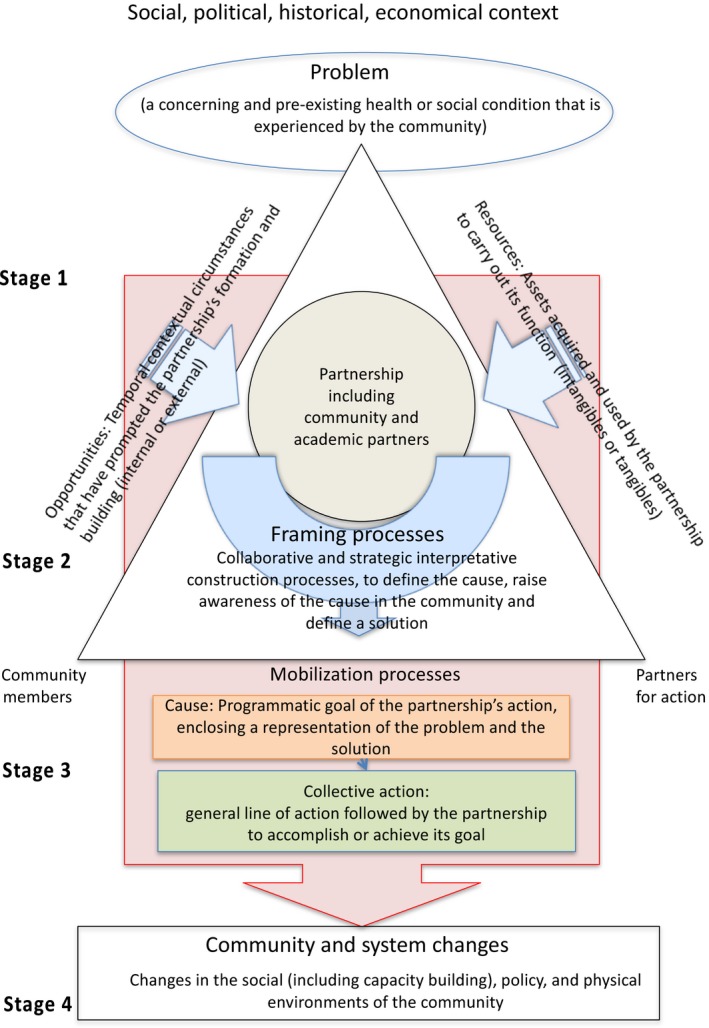
The revised conceptual framework proposes a clear picture of CBPR mobilization processes, highlighting key themes and relationships between concepts.

#### Problem (New Theme)

Although closely related to the context and a partnership's cause, the problem experienced by the community emerged as a theme in and of itself. Based on our analysis, a problem is a pre‐existing health or social issue that is experienced by the community and that takes root inside a specific social, political, historical, and economic context. In the projects studied, the problem was often perceived as a given or a matter of fact, a perception that was later challenged by the partnership through framing processes. By contrast, the cause is a representation of the problem, and is consciously and purposively framed by the partnership according to a strategic goal and contextual considerations. For instance, in Bayview Hunter Point, the pre‐existing problem was the absence of large grocery stores in the neighborhood offering residents easy access to nutritious foods, such as fruits and vegetables. The community–academic partnership identified this problem, framed it in the context of the corporate dominance of the food system in the community and used an environmental justice angle to define their cause and collective action strategy (Vasquez et al., 2007). This resulted in the creation of the Good Neighbor Program, a voluntary community food security program fostering the availability of fresh and healthy foods at affordable prices in the neighborhood.

#### Partnership (Initially: Organizational Base)

Just as the main organizational base in social movements is a social movement organization, the central structure in a CBPR project is a community–academic partnership. Indeed, based on our analysis, it is clear that the partnership is the central player and catalyzer of the mobilization process, around which everything else develops. A partnership usually begins between partners from academia and the community, but often expands to include other partners and community members in the action phase. While the academic partners are mostly researchers from different types of institutions (universities, public health agencies), community partners represent a range of groups and institutions, such as a pre‐existing community organization (as in the Latino Health for All Coalition) (Fawcett et al., 2013), a coalition of organizations (such as in the Food security project in Round Valley and in the Vietnamese REACH for Health Initiative (VRHI) Coalition) (Jernigan et al., 2012; Nguyen et al., 2006), a local health department (the Good Neighbor Program) (Vasquez et al., 2007), and a group of grassroots community members or youth recruited to form a sponsored organization (The Long Beach HOPE project and the Earth Crew Project; Cheatham‐Rojas & Shen, 2003; Vasquez et al., 2007). Notably, some partnerships successfully involved individuals and groups who were initially opponents. That is the case for the West Oakland Environmental Indicators Project, which addressed the neighborhood's disproportionate exposure to diesel truck air pollution by mobilizing truckers (among others) around the development of a truck route ordinance (Gonzalez et al., 2011).

In the context of CBPR, communities involved in partnership usually live in the same geographical area and share common characteristics be it age (youth), cultural identity, or socioeconomic status. These communities most often experience some form of marginalization that pushes them to community action to bring about social change. For instance, in the Long Beach HOPE project, the community involved consisted of immigrants experiencing racism, working in low‐paying jobs in unsafe working conditions, and experiencing high poverty and welfare rates (Cheatham‐Rojas & Shen, 2003). There are many community‐based organizations that foster social change and capacity building without academic partners (see, for instance, WE ACT, the community partner in the Earth Crew project, or Asian Communities for Reproductive Justice, which was the community‐based organization involved in the Long Beach HOPE project). However, in the context of this study, we limited our analysis to CBPR projects building on partnerships comprising at least one academic and one community partner.

Community‐based participatory research projects included in our analysis do not build heavily on established leaders or spokespersons, perhaps because of the underlying epistemology of this particular approach to research, which involves the creation of democratic and equitable relationships among parties. In the projects studied, some partners played a role only in the implementation (action) stage. These partners included different kind of entities, for instance, schools, health organizations, faith communities, youth and sports organizations, local stores, medias, as well as local policy makers.

#### Cause

In our analysis, the concept of “cause” appears to be a relevant theme to CBPR mobilization processes. Similar to the concept of cause in social movements' causes, CBPR causes are collaboratively defined by the partners through framing processes. They concurrently encompass a representation of the problem and a representation of the solution to address the problem. Most of the time, a cause reflects the main goal of the partnership's action in a simple and strategical manner, and provides impetus for community mobilization. For instance, in the West Oakland Environmental Indicators Project, the stated cause is “addressing the neighborhood's disproportionate exposure to diesel truck air pollution” (Gonzalez et al., 2011). This simple statement clearly conveys that West Oakland is more exposed to diesel truck air pollution than other neighborhoods, which poses a problem that needs to be acted on.

Causes vary from very narrow goals targeting specific health determinants, such as reducing diabetes and cardiovascular disease among Latinos in the Latino Health for All Coalition project (Fawcett et al., 2013), to broad objectives targeting general health improvement, such as promoting healthier lifestyles in the New Castle community (Minkler, Vasquez, Warner, Steussey & Facente, 2006).

#### Collective Action Strategy

The concept of collective action strategy can be thought of as the general line of action followed by the community–academic partnership to achieve its goal (cause). As in the field of social movement, this includes a variety of strategies, sometimes used in combination. Most of the included projects used a collective action strategy to address systemic determinants of health, such as the social, physical, institutional, and political causes of specific health conditions. Remarkably, almost all projects included policy advocacy at various levels (community, city, regional) to achieve their goal. For instance, the Vietnamese REACH for Health Initiative used a variety of advocacy strategies at the state level to successfully restore the Breast and Cervical Cancer Control Program in a culturally appropriated site (Nguyen et al., 2006). Less common were projects that targeted individual determinants of health, such as behaviors, beliefs, and knowledge, using educational interventions in combination with systemic strategies.

#### Framing Processes (Initially: Interpretative Frame)

Based on our analysis, the concept of “interpretative frame” was changed to “framing processes” to account for the various interpretative construction processes to which it refers. In fact, it is clear that in CBPR, there are typically many interpretative processes involved in either elucidating a new representation of a taken‐for‐granted situation, labeling a problem and defining a way to resolve it (cause), or in mobilizing community members around this solution. This is highly similar to the role played by framing processes in the context of social movements: they enable the definition of an unjust or problematic situation, the articulation of a solution and the mobilization of adherents to take action.

Framing processes in social movements often reveal inequalities and capitalize on a moral struggle, and place an emphasis on specific values and ideals, which is also the case in the context of CBPR. For instance, the Earth Crew project initially framed air pollution as an environmental justice issue: “City‐wide benefit of public transportation services is Northern Manhattan's burden” (Vasquez et al., 2006, p. 103). Then, one of the solutions implemented by the partnership involved initiating a legal complaint against a public agency; in this context, the problem was reframed as in the context of racial discrimination: “Charging the [Metropolitain Transportation Authority] with siting diesel bus depots and parking lots disproportionately in minority neighborhoods in Northern Manhattan, WE ACT and its collaborators invoked Title VI's prohibition of racial discrimination (…)” (Vasquez et al., 2006, p. 106). However, framing processes in CBPR also take into account strategic and practical considerations in line with the general context of the action. In the Long Beach HOPE project, for example, the partnership framed the problem of sexual harassment using a structural, social, and environmental lens, instead of an individual one (“the personal is political”; Cheatham‐Rojas & Shen, 2003, p. 125). The solution was advocated in terms of student safety with a gender focus to meet the schools' strategic priorities, in the context of heightened concern over school safety as a result of the Columbine school shooting: “[Sexual] harassment was thus framed as an issue of school safety for girls (…)” (Cheatham‐Rojas & Shen, 2003, p. 130). Most of the projects we analyzed illustrated a high degree of coherence between the frame used to define a representation of the problem and the frame used to articulate or advocate the solution, and considered crucial elements of context. In fact, the importance of the social, political, historical, and economic context as well as of opportunities—including funding opportunities—in framing processes cannot be ignored. For instance, it might be no coincidence that the Earth Crew project, which was largely funded by the National Institute of Environmental Health Sciences, tackled air pollution as a problem and framed it according to an environmental justice angle.

#### Opportunities

Based on our analysis, opportunities in CBPR mobilization processes appear to be deeply rooted in the context of the emerging partnership. Thus, opportunities are sometimes difficult to distinguish from resources, but should be understood as temporal contextual circumstances that have prompted the partnership's formation and development. In social movement theories, opportunities are often conceptualized as shifts and changes in the political context, emphasizing the contingency of this concept. Opportunities in CBPR can be seen as internal or external, i.e., intrinsic to the partnership history or pertaining to the external context. In the studied projects, internal opportunities consisted mostly of former relationships/collaboration between the two main partners or between one partner and the community. For instance, in the West Oakland Environmental Indicators Project, previous collaboration between the two partners laid the groundwork for the CBPR project (Gonzalez et al., 2011). In our analysis, examples of reported external opportunities consisted mostly in funding opportunities which led to the creation of the partnership and influenced the identification and framing of the problem and the cause. Remarkably, opportunities are not described in publications as playing a major role in the context of CBPR and this might be typical of research, where funding is acknowledged but its specific influence on the focus of the work is not discussed in depth. By contrast, in social movements, timing, structural, and political shifts appear as crucial to the formation of the movement, the mobilization of adherents, and its influence on the broader public agenda (Horn, 2013). In CBPR, opportunities are not frequently identified by authors as being determinant to the partnership formation. Remarkably, in the Good Neighbor Program partnership, the authors discuss preliminary developments that helped to lay the foundation for the partnership (Vasquez et al., 2007). For instance, there was already some community organizing work around environmental pollution issues and participatory research initiatives in the community, which paved the way for the project. Moreover, the municipality was seeking to prioritize redevelopment and address food insecurity through environmental justice programs; the community partner (Literacy for Environmental Justice) was created and supported by the health department partner with this aim (Vasquez et al., 2007).

#### Resources

In a CBPR context, resources are acquired and brought by academic and community partners. In CBPR, the resource mobilization process is similar to the one in social movement, which consists in mobilizing tangible or intangible assets brought by groups and individuals in the movement. In the studied projects, resources consisted mostly of intangible assets, such as expert, technical, or professional skills, or knowledge from the academic and community partners. This is not surprising given the closely intertwined action and research components of a CBPR project, which necessitate a significant set of competencies as well as expert and experiential knowledge. In the Earth Crew project, highly specialized knowledge and technical skills were required to monitor exposure to air pollution in the neighborhood (Vasquez et al., 2006). Significantly, research and its results seem to be often used as resources in and of themselves, to raise awareness of the cause in the community, and to better decide on a solution, as in the previously mentioned project (Vasquez et al., 2006). Because of the highly contextualized nature of CBPR, another equally important resource is deep experiential knowledge of the problem, the community, or the local context, which helps ensure relevance in designing and implementing the intervention. This resource is more likely to come from the community partners, who can provide crucial input in identifying key social and environmental factors affecting their health, as well as community strengths and leverage to articulate a solution. In addition, pre‐existing networks, relationships, and credibility of partners are important resources to facilitate acceptance and implementation of the action. In the Vietnamese REACH for Health Initiative, the fact that the community–academic research organization had already worked with many health organizations from the community greatly facilitated the creation of a coalition of partners (Nguyen et al., 2006). In this project, local assets in the community, such as the media, religious institutions, and other community organizations, constituted important resources that were used to increase reach and organize the action (Nguyen et al., 2006). Finally, our analysis points to funding as an essential and tangible resource; all included CBPR partnerships were funded (mostly with research grants) to support the research component of their partnership.

#### Community and System Changes (Initially: Policy, Social, or Cultural Changes)

Direct outcomes achieved by CBPR partnership are often expressed in terms of system changes (including social, physical environment, policy, and accessibility changes) in the studied projects, congruently with the cause and collective action strategy. In CBPR, system changes seem to be more easily reported than individual‐level changes because they are more proximal to the partnership's action, more easily attributed and measured. System changes are deemed to affect the environment in which individuals make choices; enabling, reinforcing, and predisposing healthy behaviors (Green & Kreuter, 2005). For instance, with the help of 40 community partners, the Latino Health for All Coalition achieved a variety of environmental changes targeting different health behaviors and determinants, including the creation of community gardens (healthy nutrition), the implementation of a new soccer program for youth (physical activity), and tree planting as well as an expanded health fair with screening for diabetes and referrals to safety‐net clinics (access to healthcare)(Fawcett et al., 2013).

However, the most significant change emphasized by authors during the interviews and in the project publications was increased community capacity to address health and social issues. This includes an increased capacity to acquire resources to develop new projects, to train and involve community members, and to tackle new and different issues, resulting in further social change. For instance, in the Food security project in Round Valley Indian Reservation Community (Jernigan et al., 2012), after successfully addressing issues related to food insecurity, the coalition garnered broader engagement from the community to develop new projects. After the academic left, community partners (without outside support) wrote a grant and secured substantial funding to address substance abuse, which was identified as a pressing issue in the community. The grant allowed them to engage in culturally appropriate substance abuse work. Thus, the initial work of the coalition built capacity and provided synergy for additional work.

#### Lifecycle of CBPR Projects (Stages 1–4)

Based on our analysis, the dynamic lifecycle of CBPR appears comparable to those of social movements. In most of the included projects, a similar process was followed, putting into perspective all the previously examined themes.

The *first stage* often starts with the creation of the partnership, usually with the specific aim of working on a pre‐existing health or social issue (problem), with the help of resources and opportunities. During the first stage, research is frequently used to document the issue of interest. This is not always the case, however. For example, the Round Valley project, started with the creation of the partnership and then engaged in a participatory prioritization process to identify the most important issues to address in the community (Jernigan et al., 2012). Sometime, the issue had already been identified and framed as a cause by a community‐based organization seeking to collaborate with an academic partner to gather more resources in the form of research expertise, knowledge, as well as funding. This was the case, for instance, of the Earth Crew project, where the community partner, WE ACT, was involved in policy advocacy on environmental issues long before its partnership with Columbia University.

During the *second stage*, the partnership defines more specifically its goal (cause) and develops a collective action strategy in view of this particular objective (system changes). This phase may involve raising awareness of the cause in the general population (sometimes by disseminating baseline research results, as in the New Castle project; Minkler et al., 2006) and mobilizing further partners and community members in defining or validating an acceptable action plan. One CBPR project, the Earth Crew project, also used policy‐oriented research during this phase to identify targets, allies, and opponents, and to develop an appropriate policy target and policy advocacy strategy (Vasquez et al., 2006).

The *third stage*, the “movement's moment,” is almost always characterized by the implementation of a multidimensional collective action strategy, with the help of the partners for action. For instance, in the West Oakland Environmental Indicators Project, this period was characterized by the implementation of many interventions to prompt the adoption by the city of the recommended policy (Gonzalez et al., 2011). In the Latino Health for All Coalition, 29 priority strategies have been planned and implemented by the action committees and community partners. A paid community mobilizer has helped to stimulate the engagement of community members and supported the implementation of these community‐determined strategies.

The *last stage* of the CBPR mobilization process can be viewed as the continuity of the partnership's action after it has achieved its goal or after the formal end of the partnership, presenting different ways by which the partnership can sustain its work. Different forms of sustainability include forming a new incorporated organization whose mission is to continue the partnership's work or to enlarge its initial scope, integrating the partnership's priority activities into partner organizations' programs, scaling up the implemented program to other levels of action, or furthering participation of the community partners in similar initiatives at higher levels of action. For instance, the Good Neighbor Program inspired a 2006 law establishing a statewide Healthy Food Purchase pilot program to improve the supply of healthy choices in small corner stores (Minkler et al., 2008). This initiative also laid the ground for other initiatives in other parts of the city (e.g., Health Retail San Francisco in the Bayview and Tenderloin area of the city; Minkler, Falbe, Hennessey Lavery, Estrada & Thayer, In press). The Vietnamese REACH for Health Initiative (VRHI) Coalition built community capacity to mobilize and bring about further changes and projects (focusing, for instance, on breast cancer screening, colorectal screening, and tobacco use) (Liao et al., 2010; Nguyen, Luong, Lehr, Marlow & Vuong, 2016; Nguyen et al., 2009). HOPE youth members have given birth to Khmer Girls in Action, an independent and autonomous community‐based organization that aims at building “a progressive and sustainable Long Beach community that works for gender, racial and economic justice led by Southeast Asian young women” (Khmer Girls in Action, 2017).

### Refined Conceptual Framework

The revised conceptual framework (Fig. 3) proposes a clear picture of community change processes in the context of CBPR, highlighting key themes and relationships between concepts. This final framework, which highlights similarities between CBPR and social movement processes, pinpoints:

- The pre‐existence of a social or health condition in the community;
- The pivotal role of the initial partnership between community and academic partners (later involving other community members and partners for action) in piloting the full mobilization process;
- Specific types of resources (intangible and tangible) and opportunities (internal and external) as necessary components feeding the partnership's development and work;
- The importance of framing processes, which encompass all the partnership's work to elucidate a new representation of a taken for granted situation, label a problem, and define a way to resolve it (cause), or mobilize community members around this solution;
- A high degree of alignment between the framing of the problem, the cause (partnership's goal), the collective action strategy, and the achieved community and system changes;
- A surrounding context that has influence throughout the process.

## Discussion

In a review on participatory research, Cargo and Mercer (Cargo & Mercer, 2008) identified three different drivers for CBPR: (a) translating knowledge into action; (b) social and environmental justice; and (c) self‐determination. Each of these drivers justifies a social movement‐derived conception of community change to act on the root and systemic causes of health inequalities. Our framework allows CBPR community change processes to be addressed in practical terms, with the added advantage of providing a temporal perspective on the development of these processes. Although not in a prescriptive sense—which would be contradictory to the fundamental assumptions of CBPR as a coconstructed process between partners—these results provide valuable theoretical guidance to researchers, intervention developers, and community actors by clarifying and detailing how mobilization processes, and consequent community and system changes, emerge and develop. At each stage of a CBPR project, the constructs of the framework can be translated into questions to guide practice and evaluation (Table 4).

**Table 4:** Guideposts for CBPR practice

Stages	Questions
1	What is the pre‐existing health or social problem that is experienced by the community? (e.g., the general health status of the community, a specific health condition or disease, a problematic health behavior, a problematic health determinants or exposure) What are the elements of the context to take into consideration relating to this problem? (Social, political, historical, economical context) Could research be relevant to document this problem at this stage? Who are the parties interested by this problem, who could be the principal partners, and how the partnership could be formalized (structure)? What are the opportunities that could be used to build the partnership? (Internal opportunities: e.g., former relationships or collaboration between the partners) (External opportunities: e.g., funding opportunities)? What are the pre‐existing resources that could be used or acquired by the partners to build the project? (Intangible resources: e.g., expert, technical, professional skills and knowledge, previous experience of the problem, research results, the community and the local context, pre‐existing networks and relationships, credibility of partners, local assets of the community) (Tangible resources: e.g., funding, office, material)
2	What frame and strategy will be used to define the cause, raise awareness, and mobilize partners and community members? o What values are foundational to the partnership or the partnering organizations, the community members? o What are the elements of the context to be taken into consideration? (Social, political, historical, economical context) o Could research results be useful to raise awareness of the cause in the community?
	What is the cause to be addressed by the partnership? What frame and strategy will be used to define the collective action? o What frame has been used to define the cause and to raise awareness? o What values are foundational to the partnership, the partnering organizations, and the community members? o What are the elements of the context to be taken into consideration? (Social, political, historical, economical context) o Could research help in defining a collective action strategy at this stage? o What other partners can be mobilized to help in defining a collective action strategy?
3	What is the stated collective action strategy of the partnership? What actions are relevant to achieve this strategy? At which levels? (Systemic/environmental level: i.e., addressing social, physical, institutional and political, determinants of health, disease, or health condition) (Individual level: i.e., addressing individual determinants of health or health conditions, such as behaviors, knowledge, beliefs) Could research help in defining and implementing alternative action strategies at this stage? What are the elements of the context to be taken into consideration when implementing the collective action? What other partners could be involved in implementing the collective action? How can other partners and community members at large be mobilized by the collective action strategy? How will system changes produced by the partnership's work be assessed?
4	What system changes have been achieved as a result of the partnership's action? 1 (i.e., social changes, physical environment changes, policy changes) Has the partnership's action evolved and continued after the formal end of the partnership? How will sustainability of the partnership's work be ensured after the end of the formal partnership? 1 (i.e., forming a new incorporated organization, incorporating the partnership's activities into partner organizations' program, scaling up the action to other levels of action with different partners, furthering participation of the community partners in similar initiatives at higher levels of action)

Table 4 is not an exhaustive list of what has to be considered when implementing or assessing CBPR. Rather, it should be viewed as a tool, informed by social movement theories, to chart the way of community change in the context of CBPR, providing useful clues and guideposts for improving CBPR practice and evaluation. Thus, we suggest that practitioners or researchers interested in applying this framework to answer programmatic needs or to find evaluation measures keep in mind that its scope refers mainly to community change processes. As such, the proposed framework is better suited to examining processes, rather than evaluating impact. In addition, the causal chart on which our framework is based ends with community and system changes, conceived as intermediate outcomes of community health improvement, as these kinds of outcomes are more easily assessed and attributed to the partnership's action (Roussos & Fawcett, 2000). Although it may seem limiting, these works are nonetheless fundamental to understanding the processes that link a CBPR partnership to meaningful community outcomes as well as the variables that affect such change processes (Roussos & Fawcett, 2000).

Validation of the analysis with key actors involved in the studied partnerships pointed to some limits to our framework. Interestingly, some participants thought that our framework did not place enough emphasis on power sharing, limiting the ability to guide and assess the development of equitable partnerships. This points to the relevance of adding a question at each stage of the mobilization process to assess who is involved, and who has the most influence. Another concern raised by some key actors interviewed was that the framework is mostly project focused, whereas many of the partnerships that form through projects are sustained after the end of the funded period. These partnerships tackle new problems with new projects, evolve into spins off, and produce additional change that is not taken into consideration by our framework. In sum, the capacity that is built in the development and implementation of projects has the potential to contribute to sustainable and continuous social change. This stresses the importance of considering the whole body of work, as well as the empowering and cascading effects of partnerships when assessing outcomes.

## Limitations

Our review is not without limitations. As mentioned earlier, the search strategy was not meant to be exhaustive, but rather focused on finding information‐rich examples of illustrative CBPR projects. We therefore developed the search strategy using the term “community‐based participatory research,” and excluded studies using “participatory action‐research,” “participatory research,” and other related terms. This choice, which builds on practical considerations, has potentially excluded other relevant projects. Thus, the context‐specific framework developed from the review is highly relevant in the context of our larger project, but perhaps not generalizable to all participatory research projects.

Furthermore, the initial framework and related themes were generated from theories identified as relevant to the review question, but another review team could have identified other relevant theories and themes, leading to slightly different analysis and results. In fact, the interpretation of results is limited by the use of our framework. However, we have made the rationale for this choice of framework transparent. In addition, the secondary thematic analysis, inherent to framework synthesis, mitigates this limitation by providing opportunities for new themes, concepts, and categories to emerge from the data, thus encompassing specificity of the setting, population, or intervention that can fall outside of the foundation framework (Carroll et al., 2013).

Finally, a limitation of this type of research is that it draws mostly on what has been published on the projects selected, thus excluding developments (sometimes the most interesting) that are not captured in peer‐reviewed scientific papers. To address this limitation, we conducted interviews with key actors involved in the CBPR projects studies to validate our findings and collect additional data.

## Conclusion

Framework synthesis, building on social movement theories, has proven to be a useful analytic strategy to conceiving and mapping community change processes in the context of CBPR. The resulting revised framework that draws on existing theoretical foundations provides a context‐specific and evidence‐based model to generate a new, innovative understanding of these processes. It is relevant to CBPR projects sharing fundamental principles, but implemented in numerous settings, and with different types of partners and a broad range of goals. Our framework provides valuable practical guideposts for CBPR practice and evaluation by clarifying and detailing how mobilization processes and consequent system changes emerge and develop from CBPR.

## Conflict of Interest

The authors have no conflict of interest to declare.

## Appendix 1 Included CBPR projects

**Table:**

	Main document	Companion papers and related documents	Name of the intervention/partnership	Website
1	Cheatham‐Rojas and Shen (2003)	0	The Long Beach HOPE (Health, Opportunities, Problem‐solving and Empowerment) project (Long Beach, California, US)	Project's spin‐off website (Khmer girls in action): http://kgalb.org
2	Fawcett et al. (2013)	5 (scientific articles)	The Latino Health for All Coalition (LHFA), (Kansas City, Kansas, US)	https://www.myctb.org/wst/latinohealth/default.aspx
3	Gonzalez et al. (2011)	8 (4 scientific articles, 4 reports)	The West Oakland Environmental Indicators Project (WOEIP) (West Oakland, California, US)	http://www.woeip.org
4	Jernigan et al. (2012)	2 (scientific articles)	The Round Valley Community Coalition (Round Valley Reservation, Northern California, US)	
5	Minkler et al. (2006)	3 (1 scientific article and 2 reports)	The Healthy Cities Committee of New Castle Partnership (New Castle, Indiana, US)	Incorporated community partner organization website (Healthy Communities of Henry County): http://www.hchcin.org
6	Nguyen et al. (2006)	20 (17 scientific articles and 3 reports)	Vietnamese REACH for Health Initiative (VRHI), (Santa Clara County, California, US)	
7	Vasquez et al. (2006)	5 (2 scientific articles, 2 book chapters, 1 report)	The Earth Crew project (West Harlem, New York, New York, US)	Community partner website: http://www.weact.org/history
8	Vasquez et al. (2007)	7 (3 scientific articles, 1 dissertation, 1 book chapter, and 2 report)	The Good Neighbor Program (Bayview Hunters Point, San Francisco, California, US)	Community partner website: http://www.lejyouth.org/about-us/
Total	8 main papers	50 companion papers and project‐related documents		

## Appendix 2 Distilled summaries of the included projects

**Table:**

No	Name of the intervention/partnership	Context	Problem	Original partnership	Cause	Collective action strategy
1	The Long Beach HOPE (Health, Opportunities, Problem‐solving and Empowerment) project (Long Beach, California)	Community of approximately 30,000 Cambodians, characterized by a lack of political involvement, low‐paying jobs, unsafe working conditions, and high poverty and welfare rates. The Cambodian community is still suffering from impacts of war and violence in Cambodia and racial profiling in the welfare system	A high rate of sexual harassment in youth	Between a community‐based organization (Asian Communities for Reproductive Justice) including some academic members (postdoc) and community members (HOPE members, i.e., young Cambodian girls)	To involve youth as agents of change in addressing sexual harassment in youth	Empowering youth in addressing systemic and individual challenges to sexual harassment in youth
2	The Latino Health for All Coalition (LHFA), (Kansas City, Kansas)	Latino residents of Wyandotte County (26.3%), Kansas, mostly first‐generation, low‐income, uninsured, and with low levels of education. Principally of Mexican descent (81%). Latinos living in Kansas City have a life expectancy 11 years shorter than Whites, and are nearly 1½ times more likely to die from diabetes	A high prevalence of diabetes and cardiovascular diseases and related risk factors of unhealthy diets, physical inactivity, and limited access to health services	Between academic partners (University of Kansas's Work Group for Community Health and Development and Juntos Center for Advancing Latino Health at the Kansas University Medical Center) and a community organization (El Centro). (Was later extended to include another 40 + organizations, including community organizations, government agencies, and faith‐based institutions)	To reduce diabetes and cardiovascular disease by promoting healthy nutrition, physical activity, and access to health services among Latinos in Kansas City/Wyandotte County	Drawing on the Health for All model, promote environmental changes through targeted, community‐determined and ‐led interventions at different ecological levels, which will result in behavior changes and consequently improved health outcomes for the community
3	(WOEIP) (West Oakland, California)	A vibrant community (approx. 22,000 persons) of predominately low‐income African American and Latino residents located on the San Francisco Bay. Bounded by freeways, this community is exposed to thousands of moving and stationary sources of diesel pollution. Important background of social activism in the neighborhood	High youth asthma rates and diesel truck traffic in the neighborhood of West Oakland	Between a research partner (the Pacific Institute) and a community organization (West Oakland Environmental Indicators Project)	To address the neighborhood's disproportionate exposure to diesel truck air pollution	Addressing, through a range of intersecting policy and advocacy efforts, disproportionate pollution exposure in the neighborhood (environmental changes) and increase community participation in decision making
4	Food security in Round Valley Indian Reservation community (Northern California)	Round Valley is a rural and isolated Indian Reservation in Mendocino County, in Northern California (approx. 4000 persons). The community is characterized by low education and income levels, high unemployment, high rate of obesity and diabetes, but also by progressive values, and community activism. A third of families receive food from a food distribution program. There is no nearby supermarket, only one local grocery store (selling overpriced and packaged foods) and a gas station that sells food. There is a lack of Native‐owned shops or stands at the farmers' market	Food insecurity in the rural Indigenous Reservation of Round Valley	Between an academic partner (the University of California) and a coalition of community organizations (including community leaders, Round Valley Indian Health Center staff, California Indian Health Service representatives)	To identify and address upstream causes of food insecurity in a rural California reservation	Addressing systemic barriers to food access through policies and interventions
5	Partnership with the Healthy City Committee of New Castle (New Castle, Indiana)	New Castle is a rural town whose automobile industry was central to the economy. The population has declined in recent decades, as a result of declines in the automobile industry. The community uses to help itself and utilizes the resources available; the pre‐existing Healthy City Committee is an example. The community is characterized by a conservative mindset	Low individual health indicators and health behaviors in New Castle, including high rates of smoking, problematic dietary choices, low physical activity, and other	Between an academic partner (Indiana University's School of Nursing) and a community organization (the Healthy City Committee of New Castle)	To promote healthier lifestyle in New Castle	“Making the healthy choice the easy choice” through a variety of environmental (‘small p policy’) changes.
6	Vietnamese REACH for Health Initiative (VRHI) Coalition, (Santa Clara County, California)	The Vietnamese community of Santa Clara County, California (102,841) is a large community with churches, pagodas, stores, restaurants, health providers, and community‐based organizations. Almost all Vietnamese‐Americans arrived as refugees after 1975, when the Vietnam War ended, and resulted for many in changes in socioeconomic status. Many households remain linguistically isolated. The main barrier to health remains access to affordable and culturally appropriate care. This community has many low‐income immigrants, but also an educated and acculturated pool of professionals with a strong ethnic identity	A high rate of cervical cancer in Vietnamese‐American women in general	The Vietnamese REACH for Health Initiative (VRHI) Coalition which was formed through a partnership between a community–academic research organization (Vietnamese Community Health Promotion Project, University of California) and a number of community organizations (including the Department of Public Health, health care organizations or organizations serving low‐income or recent Vietnamese immigrants, and community leaders)	To increase cervical cancer awareness and screening (Pap test) and, more generally, to improve the health of the whole community of Vietnamese‐Americans of Santa Clara County	Addressing individual, systemic (providers and health care system) and environmental (financial and cultural) barriers to cervical cancer screening
7	The Earth Crew project, Partnership between West Harlem Environmental Action (WE ACT) and Columbia University (West Harlem, New York)	West Harlem is part of Northern Manhattan, comprising 627,000 low‐income to mid‐income African‐Americans and Latinos. The community has a rich and diverse population and cultural history, but disproportionate rates of disability and premature death (one in four children suffer from asthma). Six of the eight Manhattan diesel bus depots housing were sited in Northern Manhattan	A high rate of asthma, and related morbidity and mortality in the neighborhood of West Harlem	Between an academic partner (Columbia University's Children's Center for Environmental Health) and a community organization (West Harlem Environmental Action)	To address the neighborhood poor air quality	Addressing air pollution environmental injustice through policy changes and community leadership development
8	The Good Neighbor Program (Bayview Hunters Point, San Francisco, California)	Bayview Hunters Point is an economically disadvantaged neighborhood of San Francisco, populated by African Americans, Asian Americans, and Pacific Islander Americans (30,000 persons). The environment is considered as a food desert, characterized by a lack of access to culturally appropriate, affordable, and healthy food, and making it difficult for local residents to access nutritious foods such as fruits and vegetables	Food insecurity in Bayview Hunters Point's Neighborhood	Between a community organization (Literacy for Environmental Justice), a consultant academic partner (research evaluator) and a local health department (San Francisco Department of Public Health)	To increase the access to healthy food in the neighborhood (food security), and to decrease the display of and access to tobacco products	Addressing the link between tobacco and food security and increase community accessibility to healthy food with policy and environmental efforts

No	Name of the intervention/partnership	Framing processes	Opportunities	Resources	System and community changes achieved	
1	The Long Beach HOPE project (Long Beach, California)—Health, opportunities, Problem‐solving, and empowerment project	The partnership framed sexual harassment from a structural, social, and environmental viewpoint, instead of an individual one: “The personal is political” (Cheatham‐Rojas & Shen, 2003, p. 125). The solution was advocated in terms of student safety with a gender focus to meet the concern of local actors from schools. “Sexual harassment was thus framed as an issue of school safety for girls (…)” (Ibid, p. 130)	Development of HOPE projects for Southeast Asian girls by the community organization (ACRJ) catalyzed the project (the Long Beach HOPE project was one of the two)	Technical, professional skills in the ACRJ staff; Experience from the ACRJ staff in community organizing; Support for youth participation; Experiential knowledge from the HOPE members; Direction and action provided by youth in the community; Funding (e.g., stipend for participation; funding for project)	District‐wide school policy changes including educational sessions provided at schools around sexual harassment, training to school interveners, improved grievance procedure to monitor and address incidents. This project has allowed to build capacities of the community partner. Khmer Girls in action, an independent community‐based organization, is an offshoot of this project. HOPE is now a vibrant community organization and plays an important role within the larger reproductive justice community	
2	The Latino Health for All Coalition (LHFA), (Kansas City, Kansas)	Health was framed as a social and complex issue with multiple causes. The resulting program was developed around different levels of interventions, targeting different behaviors and their social determinants, and taking into account a shared Latino culture	Earlier participatory work around chronic disease prevention in Kansas City and testing of the Health for All Model of action laid the groundwork. This project developed in the context of a specific funding program of the National Institutes of Health (NIH)	Experience from a previous academic–Community Partnership in Kansas City (Health for all model); Skills and knowledge from academic partners; Skills, knowledge of the local context from the community partner; Existing tools to build community capacity and to monitor and evaluate activity and communicate (Community Tool Box and the Action Tool Kit); Funding, including a grant from the National Institute on Minority Health and Health Disparities, National Institutes of Health	A variety of physical, social, and environmental changes have been achieved at the individual, family, organizational, and community levels. The changes include (for instance) the creation of community gardens, a new soccer program for youth, and an expanded health fair with screening for diabetes and referrals to safety‐net clinics. The Coalition has been sustained and they have continued to be funded	
3	West Oakland Environmental Indicators Project (WOEIP) (West Oakland, California)	Diesel truck traffic was first framed as a safety and environmental justice health concern, but the problem was strategically framed as a health issue (instead of a traffic, or walkability one) when advocating for a solution (truck route ordinance) to local policy makers. “We could have said the truck route was about traffic. We could have said it was about walkability in the neighborhood. We could have said it was about a whole lot of things [but] we said it was about health” (Gonzalez et al., 2011, p. S169)	An initial collaboration between the two partners laid the groundwork for a CBPR project. The collaboration's work drew attention from local media and the community. Then, the partnership received funding from the federal Environmental Protection Agency and the California Department of Health Services	Expertise, skills, and knowledge from the academic partner; Technical expertise from a consulting firm; Experiential knowledge from the community partner; Policy‐maker allies; Funding from many private foundations (Bank of America Foundation, Firedoll Foundation, Wallace Alexander Gerbode Foundation, William and Flora Hewlett Foundation, Malcolm Pirnie, Inc., The San Francisco Foundation, and the Wells Fargo Foundation)	Policy change, mainly a city ordinance for the implementation of a truck route in the neighborhood. There was also an increased community capacity to involve and train people in CBPR. Subsequently to the project, the partnership secured $22 million from the EPA to retrofit or replace old trucks and they significantly reduced pollution over a 5‐year period	
4	Food security in Round Valley Indian Reservation community (Northern California)	Food insecurity was first framed as a racial, structural, and environmental issue. “[Community members] pointed out that the absence of Native‐owned stands at the weekly farmers' market as well as its location in a mostly white area of town made Native people feel unwelcome.” (Jernigan et al., 2012, p. 650). The racial justice angle, which was seen as an “overwhelming and somewhat abstract” issue (Ibid, p. 650), was evacuated to focus on more actionable factors.	The project developed thanks to funding from different agencies and the process was led and facilitated by the principal investigator	Knowledge and expertise from academic partners; Leadership, experience, knowledge, and credibility of community leaders; Funding for the project itself and for specific miniprojects from the Robert Wood Johnson Foundation New Connections Active Living Research program, California Endowment, and California Department of Transportation	Social, physical, environmental, and policy changes including the creation of a Producers' Guild, which foster an integrated community supported agriculture and commodity food program, the addition of Electronic Benefits Transfer machine at the local market, the introduction of culturally appropriate foods at the local market and inclusion of vegetables, fruits, and food for people with diabetes at the grocery store. After successfully addressing issues related to food insecurity, the coalition garnered broader engagement and conducted more formal training. The initial work of the coalition built capacity and provided synergy for additional work	
5	Partnership with the Healthy City Committee of New Castle (New Castle, Indiana)	The health status (problem) of the community was framed in terms of deficit (compared to national goals and norms) to raise awareness. In this “conservative and anti‐regulatory” community (Minkler et al., 2006, p. 298), the solution was implemented in incremental changes, and advocated in terms of individual rights (“make the healthy choice the easy choice” (Ibid, p. 295)) instead of policy changes	The pre‐existing community partner (Healthy City Committee of New Castle) had a mission of promoting the health of the city through multisectoral collaborations. A grant from the WK Kellogg Foundation funded the initial collaboration	Knowledge and expertise from the academic partner; Experience, skills, and network from the community partner; Initial funding from the W. K. Kellogg Foundation	Social, physical, environmental, and policy changes including a bill restricting indoor smoking in public places, an elaborated playground on City owned land, tree planting, and an initiative to develop a system of trails throughout the county	
6	Vietnamese REACH for Health Initiative (VRHI) Coalition, (Santa Clara County, California)	Cervical cancer screening was conceived of as an individual, medical, and systemic issue, with multiple pathways of intervention. “The VRHI Coalition concluded that improvements in knowledge alone would be insufficient to address the barriers, particularly financial and cultural, to Pap testing in a sustainable manner.” (Nguyen et al., 2006, p. 36) “The Logic Model for the [Community Action Plan] was based on the Pathways Model, which posits that healthy behaviors occur after successful negotiations of complex pathways, each of which contain predisposing, enabling, and reinforcing factors.”(Ibid, p. 36)	The pre‐existence of the community–academic research organization (VCHPP), which had a long history of collaboration with the community, laid the ground work for the project. The project took form thanks to a CDC planning grant	Expertise, skills, and knowledge from the Academic partner; Credibility of the community–academic research partner, VCHPP; Experience of the local and cultural context, professional skills, networks, and media of the community partners; Knowledge and resources of community members (not only leaders but also staff and lay health workers) in partner organizations; Credibility of the community members that provided reach into the community; Local assets (existing ethnic media, pagodas, CBO); Media from the partners; Funding from the Centers for Disease Control and Prevention, the Asian American Network for Cancer Awareness, Research and Training, and the American Cancer Society Cancer Control	Social, physical, environmental, and policy changes such as the restoration of the Breast and Cervical Cancer Control Program in a culturally appropriated site and the establishment of a weekly low‐cost Pap clinic staffed by a Vietnamese‐speaking female physician. The project also built capacity in the community to mobilize and bring about further changes and projects (focusing, for instance, on breast cancer screening, colorectal screening, and tobacco use)	
7	The Earth Crew project, Partnership between West Harlem Environmental Action (WE ACT) and Columbia University (West Harlem, New York)	From the start, air pollution was framed as an environmental justice issue. “City‐wide benefit of public transportation services is Northern Manhattan's burden” (Vasquez et al., 2006, p. 103). One of the solutions carried out by the partnership involved initiating a legal complaint against a public agency, and the problem was reframed as a racial discrimination one. “Charging the MTA with siting diesel bus depots and parking lots disproportionately in minority neighborhoods in Northern Manhattan, WE ACT and its collaborators invoked Title VI's prohibition of racial discrimination (…)” (Ibid, p. 106)	The existing partnership between the community (WE Act) and academic partners (Columbia University) facilitated the project	Experience form previous background work between the two partners; Expertise, guidance, and knowledge from the academic partner; Expertise, local experience, and knowledge from the community partner in advocacy and community mobilization; Credibility of the partnership's research; Strong policy alliances; Medias; Funding from the National Institute of Environmental Health Sciences (NIEHS)	Policy change, mainly a substantial impact on the conversion of the Metropolitan Transport Authority bus fleet to clean diesel, the establishment, by the Environmental Protection Agency of permanent air monitoring in Harlem and other local and national “hot spots” and the development of an environmental justice policy at a state level. From this project, there was an empowered capacity in youth to address environmental justice issue in the neighborhood. WE ACT is still partnering with community‐based youth serving organizations, which helps them leverage resources and maximize their reach	
8	The Good Neighbor Program (Bayview Hunters Point, San Francisco, California)	Formed thanks to the Tobacco Free Project, the partnership first framed food insecurity according to tobacco food subsidiary. Food insecurity was viewed as an environmental justice issue linked to the corporate dominance of the food system: “The relationship between health and the corporate dominance of the food system became an integral part of the LEJ partnership's problem definition and later policy intervention” (Vasquez et al., 2007). The solution advocated to local policy makers was strategically linked with the community improvement priority of the city. “The city‐based priorities of redevelopment and community violence also may have served as windows of opportunity to produce an ideal environment for opening the discussion about food insecurity and its connection to community improvement.” (Ibid, p. 346)	Existing community organizing work around environmental pollution and participatory research initiatives in the community, laid the groundwork for the project. Moreover, there were some efforts from the municipality to prioritize redevelopment and address food insecurity through environmental justice programs. The community organization (LEJ) was created and supported by the health departmet with this aim	Knowledge, technical expertise and assistance from the health department and evaluator partner; Networks, allies (policy makers) and experience of the health department with community actors; Funding from the San Francisco Department of Public Health's Tobacco Free Project; Four city departments contributing staff, resources, and incentives to manage and sustain the program	Physical, social, environmental, and policy changes, including the implementation of the Good Neighbor Program, a voluntary community food security program fostering the availability of fresh and healthy foods at affordable prices in the neighborhood, and the development of a city‐ and foundation‐sponsored initiative to expand the program. This initiative laid the ground for other work in other parts of the city (e.g., Health Retail San Francisco in the Bayview and Tenderloin area of the city). In 2013, a legislation that created Healthy Retail San Francisco was introduced	
